# Characterization of *Salmonella* Isolates Recovered from Stages of the Processing Lines at Four Broiler Processing Plants in Trinidad and Tobago

**DOI:** 10.3390/microorganisms9051048

**Published:** 2021-05-13

**Authors:** Anisa Sarah Khan, Karla Georges, Saed Rahaman, Woubit Abebe, Abiodun Adewale Adesiyun

**Affiliations:** 1 School of Veterinary Medicine, Faculty of Medical Sciences, University of the West Indies, St. Augustine, Trinidad and Tobago; anisakhan11@gmail.com (A.S.K.); Karla.georges@sta.uwi.edu (K.G.); 2 Veterinary Public Health Unit, Ministry of Health, Port of Spain, Trinidad and Tobago; saed.rahaman@gmail.com; 3 Department of Pathobiology, Tuskegee University College of Veterinary Medicine, Tuskegee, 201 Frederick D Patterson Dr, Tuskegee, AL 36088, USA; wabdela@tuskegee.edu; 4 Department of Production Animal Studies, Faculty of Veterinary Science, University of Pretoria, Private Bag X04, Onderstepoort, Pretoria 0110, South Africa

**Keywords:** broiler processing plants, *Salmonella*, serotypes, risk factors, antimicrobial resistance, Trinidad

## Abstract

This cross-sectional study determined the prevalence, characteristics, and risk factors for contamination of chicken with *Salmonella* at four operating broiler processing plants in Trinidad. Standard methods were used to isolate and characterize the *Salmonella* isolates. The overall prevalence of *Salmonella* at the four processing plants was 27.0% (107/396). The whole carcass enrichment (WCE) method yielded a statistically significantly (*p* = 0.0014) higher frequency of isolation (53.9%; 97/180) than the whole carcass rinse (35.0%; 63/180) and neck skin methods (42.2%; 38/90). *S.* enterica serotypes Enteritidis, Javiana, and Infantis were the predominant serotypes isolated accounting for 20.8%, 16.7% and 12.5%, respectively, of the serotyped isolates. Risk factors included the use of over 100 contract farmers (OR 4.4), pre-chiller (OR 2.3), addition of chlorine to chiller (OR 3.2), slaughtering sick broilers (OR 4.4), and flocks with >50% mortality. Multi-drug resistance was detected in 12.3% (14/114) of the isolates of *Salmonella*. Resistance was high to kanamycin (85.7%) and doxycycline (74.6%) but low to amoxicillin-clavulanic acid (2.4%) and sulphamethoxazole-trimethoprim (0.8%). The occurrence of resistant *Salmonella* in chickens processed at commercial broiler processing plants has implications for salmonellosis and therapeutic failure in consumers of improperly cooked contaminated chickens from these plants in the country.

## 1. Introduction

Salmonellosis is the third leading cause of death among food transmitted diseases [1] with an estimated global *Salmonella* enterocolitis incidence of 95.1 million cases [2], accounting for 50,771 deaths in 2017 [3]. In the Caribbean, *Salmonella* is the most common laboratory-confirmed cause of foodborne diseases since 2005 [4]. Poultry has been reported to be the main carrier of *Salmonella* infections to humans [5], more common than any other animal species [6]. Broiler meat is an economical source of protein and estimated to be the most widely consumed meat, globally.

The human population of the twin-island Republic of Trinidad and Tobago is 1,366,725 [7] with a reported 58.3 kg per capita poultry consumption rate; 800,000 broilers are produced weekly, of which 20% are imported [8]. Consumers purchase chicken from cottage poultry processors, where they are freshly slaughtered and from supermarkets, which offer both chilled and frozen locally processed or imported frozen chicken. Broiler processing plants are responsible for 50% of local broiler processing [9] where supermarkets and the franchised foodservice sector are supplied with chilled chickens as well as further-processed products [9].

Several studies have reported the high frequency of contamination with *Salmonella* of chicken meat sold at the informal and formal outlets in developed and developing countries [10,11]. It has also been reported that the processing of chicken at commercial processing plants contributes significantly to the contamination of dressed chicken carcasses with *Salmonella* before they reach the retail outlets [12,13]. Unhygienic carcass handling, soiled slaughter equipment [14,15], contaminated water (scalding and immersion chiller water), and waste generated from evisceration and the de-feathering processes have been implicated as major sources of *Salmonella* contamination during broiler processing [16,17,18]. *Salmonella*-free broilers leaving farms may potentially become contaminated by the pathogen during processing through contact with immersion chiller water contaminated with *Salmonella* originating from the positive broilers [19,20]. This can occur, should there be improper pH and chemical agents’ concentrations, as well as a failure to maintain good sanitary practices throughout processing [21].

With the increase in production and consumption of broiler meat over the years, the use, misuse, and overuse of veterinary drugs for prophylaxis, therapeutic, and growth promotion purposes [22,23] are common in countries such as Trinidad and Tobago. In the country, although regulations on the use of veterinary drugs in livestock exist, they are not routinely enforced. The increase in the isolation of *Salmonella* in humans, and the resistance of *Salmonella* strains to antimicrobial agents commonly used in food-producing animals is a major health concern [24,25]. Worldwide, of a greater concern is the emergence of multidrug-resistant (MDR) *Salmonella* [26], which has been implicated in foodborne outbreaks due to contaminated meat [27,28].

To isolate *Salmonella* from poultry processing plants, different approaches have been reported and recommended. In the European Union, the use of neck skin (NS) maceration [29] is most frequent whereas, in the United States, the U.S. Department of Agriculture Food Safety and Inspection Service (USDA-FSIS) [30] recommends the use of whole carcass rinse (WCR) method. Whilst the WCR is the most commonly used method for isolation of *Salmonella* in broiler carcasses [31,32,33], the whole carcass enrichment (WCE) and neck skin (NS) methods have been shown to be just as effective [34] or even more than the WCR [35]. However, the large space required for incubating whole carcasses makes the WCE method impractical for routine testing, but it is valuable for research purposes [36].

In Trinidad and Tobago and the Caribbean, there is a dearth of comprehensive up-to-date data on the role played by the commercial broiler processing plants in the contamination of processed chicken carcasses with *Salmonella*. The only available recent published data were from studies conducted at the outlets of cottage poultry processors (‘wet market’) where the slaughtering and retailing of dressed chicken were practiced [37] and at supermarkets where retailing of chicken from the commercial processing plants occurs [38] and the antimicrobial resistance profiles of *Salmonella* isolates from both sources were determined [39].

Considering the limited current information on the status and dynamics of *Salmonella* contamination of chicken carcasses at the commercial broiler processing plants, the present study with the following objectives was conducted: (i) to determine the frequency of isolation of *Salmonella* longitudinally from the different stages of processing, from pre-slaughter broilers to chilled carcasses, (ii) to evaluate the efficacy of three isolation methods for *Salmonella*, (iii) to identify the risk factors associated with *Salmonella* contamination of chicken carcasses at the plants and finally, (iv) to determine the serotypes and antimicrobial resistance profiles of the isolates of the pathogen recovered from the four plants operating in Trinidad.

## 2. Materials and Methods

### 2.1. Sampling Site

The study was conducted in Trinidad and Tobago, the twin-island Caribbean country located in the southern Caribbean, north-east of the South American country of Venezuela, northwest of Guyana, and south of Grenada in the Lesser Antilles. There are currently four commercial broiler processing plants in Trinidad. These plants process only broiler chickens and supply supermarkets and food outlets with dressed chilled and/or frozen chicken. Each processing plant packages whole dressed chickens, various packaged chicken parts (legs, thighs, breasts, wings, and mixed parts), offal (liver, gizzard), feet, and necks all of which are available for sale at their retail outlet (at the respective plant) or supplied to supermarkets or food outlets. The similarities and differences in the operations that may impact on the bacteriological quality of broilers at the four processing plants studied are shown in a flow chart (Supplementary data: S1, A–D).

The number of samples to be collected for this study was estimated using the formula [40]:

Estimated sample size for an infinite population,

$$n_{o}={Z_{u}}^{2}P_{\mathrm{ex}}\;(1-P_{\mathrm{ex}})/d^{2}\;$$

where:

$n_{o}$ = Estimated sample size; Z_u_ = Degree of confidence= 1.96;

P_ex_ = Expected prevalence = 50%; d = Desired absolute precision = 5%;

$n_{o}$ = [1.96^2^ × 0.5(1 − 0.5)]/0.05^2^ = 384.

A total of 396 samples were collected comprising swabs of pre-slaughter cloacae, pre-evisceration carcasses, post-evisceration carcasses, chilled whole chickens (dressed), and chilled chicken parts (dressed), as well as neck skins and chiller water. Sample collection was conducted during the period from January to September 2019. The total number of samples collected at each plant was determined using proportional sampling based on their throughputs. Therefore, two, four, one, and two sampling visits were made to plants A, B, C, and D, respectively. Plant A and D received chicken from their 210 and 98 contract farms, respectively, whereas Plants B and C were owned by the same parent company that controlled 32 farms. Samples were collected in individual sterile bags and bottles and transported on ice to the laboratory of the Veterinary Public Health Unit, School of Veterinary Medicine for processing within 4–6 h after collection. Standardized, pre-tested questionnaires were administered at each broiler processing plant to obtain information about demography, operational information, and risk factors for carcass contamination with *Salmonella*. Some of the questions were designed to elicit information on the average number of contract farmers, the average waiting period between arrival of chickens to slaughter, disposal of waste material, and source of water supply (Supplementary data: S2).

### 2.2. Processing of Samples Collected from Processing Plants

During each visit to the broiler processing plant the following samples were collected in sterile bottles/bags: 10 cloacal swabs, 5 pre-evisceration carcasses (post-defeathering), 5 post-evisceration carcasses, 10 neck skins, 4 immersion chiller water samples, 5 chilled whole carcasses (after removal from immersion chiller), and 5 packs of chilled chicken parts each of legs, thighs, breast, wings, and mixed parts.

The WCR method, described by the USDA-FSIS [30] for *Salmonella* isolation was used. Each carcass was rinsed in 430 mL of buffered peptone water (BPW) (Oxoid, Hampshire, UK), rotated for no less than 30 times and 30 mL of the rinsate was removed and incubated.

Each carcass with the remaining 400 mL BPW in the WCR process above, was incubated in accordance with the WCE method as described by Cox et al. [41] and constituted the WCE sample. Neck skin (NS) samples were processed as recommended by the Commission Regulation (EC) No 2073/2005 [42] with the following modification. Each neck skin was collected in a sterile bag from which approximately 10–15 g was aseptically excised and added to BPW in a 1:9 ratio and incubated at 37 °C for 18–24 h. Each excised neck skin was treated as one (1) sample as performed in other studies [34,43].

Each cloacal swab sample was added to 9 mL BPW and subsequently incubated [44]. During each sampling visit to the plants, 400 mL of immersion chiller water was collected four (4) times, at an interval of 1.5 h to provide representative samples of potential contamination over a 6 h period. In the laboratory, 100 mL were aseptically removed from each 400 mL sample and centrifuged at 4470× *g* for 20 min after which 1 mL of sediment was removed and transferred to 9 mL BPW and incubated [45].

All pre-enriched BPW samples were incubated at 37 °C for 18–24 h. Samples were then selectively enriched in 9 mL tetrathionate (TT) broth (Oxoid, Hampshire, UK) and 9 mL Rappaport-Vassiliadis Soya (RVS) broth (Oxoid, Hampshire, UK) and incubated at 37 and 42 °C, respectively.

### 2.3. Isolation and Identification of Salmonella

Samples enriched in selective broths were sub-cultured onto Xylose–lysine–tergitol 4 (XLT-4; Oxoid, Hampshire, UK) and Brilliant green agar (BGA; Oxoid) and incubated at 37 °C for 18–24 h. Suspected *Salmonella* colonies that displayed characteristic colonies on both selective agar plates were then purified on blood agar plates (Oxoid) and incubated at 37 °C for 18–24 h. Pure cultures were subjected to a panel of biochemical tests that included triple sugar iron agar, lysine iron agar, urea, citrate, methyl red, sulfide-indole-motility medium, and o-nitrophenyl-b-D-galactopyranoside (Oxoid) [39,46]. Isolates biochemically confirmed as *Salmonella* were then subjected to a slide agglutination test using *Salmonella* polyvalent antiserum (A-I & Vi, Difco, Detroit, MI). Complete confirmation and serotyping of *Salmonella* isolates representative of those recovered by the WCR/WCE/NS, RVS/TT, and BGA/XLT-4 methods were performed using the phase reversal technique, and the results interpreted according to the Kauffman–White scheme [47] at the Public Health Laboratory, Ministry of Health, St. Michael, Barbados. Molecular confirmation of tentatively identified *Salmonella* was conducted using conventional polymerase chain reaction (PCR). Initially, DNA was extracted from the *Salmonella* isolates by the boiling method [37,48], followed by the use of conventional PCR to detect the *inv*A gene as described earlier [37,48]. The following primer sequences were used to amplify a 284 bp fragment of the *inv*A gene, Forward: 5′ GTGAAATTATCGCCACGTTCGGGCAA 3′ and Reverse: 5′ TCATCGCACCGTCAAAGGAACC 3′ as described by Oliviera et al. [49].

### 2.4. Determination of Antimicrobial Resistance

The antimicrobial resistance of 126 *Salmonella* isolates recovered from the samples obtained at the four broiler processing plants was determined using the disk diffusion method according to the Clinical and Laboratory Standards Institute [50,51] guidelines. Eight antimicrobial agents commonly available in the local market and frequently used in the poultry industry in Trinidad formed the panel of antimicrobial agents. The antimicrobial agents, concentrations, and classes (Difco, Becton Dickinson, Sparks, MD, USA) used comprised the following: amoxicillin-clavulanic acid (AMC, 30 µg, β-lactam); doxycycline (DO, 30 µg, Tetracycline); ceftriaxone (CRO, 30 µg, Cephalosporin); gentamicin (CN, 10 µg, Aminoglycosides); kanamycin (K, 30 µg, Aminoglycosides); chloramphenicol (C, 30 µg, Phenicol); sulphamethoxazole–trimethoprim (SXT, 23.75 and 1.25 µg, Sulphonamides); and ciprofloxacin (CIP, 5 µg, Fluoroquinolones). The tests were performed on Mueller–Hinton agar (Difco), followed by aerobic incubation at 37 °C for 24 h. The zones of inhibition were interpreted as recommended by the disk manufacturer and Clinical and Laboratory Standards Institute [50].

### 2.5. Statistical Analysis of Data

Chi-square analyses were conducted using the Statistical Package for Social Sciences, SPSS (version 27, IBM Corp., Somers, NY, USA) to determine statistically significant associations in the frequency of isolation of *Salmonella* amongst (i) the three different sampling methods, (ii) the risk factors associated with *Salmonella* contamination, (iii) the types of samples collected, and iv) the plants sampled. The Fisher’s exact test was used for 2 × 2 contingency tables with expected frequencies of <5. The level of significance was set at an alpha = 0.05. Univariate analysis of associations was conducted using the *Salmonella* status of the sample as a binary outcome (positive or negative). The predictor variables were the average number of farmers, number of workers directly involved in processing, waiting period, the mortality rate on arrival, treatment of diseased birds, use of a pre-chiller, agents used in chiller, temperature of chiller water, and segregation of workers. Each predictor variable was tested for significant associations with the *Salmonella* status using the chi-square test of association. Significant variables (*p* < 0.05) in the univariate analysis were assessed for collinearity using the chi-square statistic and were considered collinear if *p* < 0.05. A forward stepwise regression model where entry of *p* < 0.5 and removal of *p* < 0.10 was used in the regression analysis. Hosmer–Lemeshow chi-square was used as a goodness of fit test. Statistical analysis was done using SPSS (version 27) at an alpha level of 0.05.

## 3. Results

### 3.1. Overview of Management and Production Data

In Trinidad, the poultry industry is vertically integrated, where each company controls its respective hatcheries, contracted farms, feed mills, and processing plant. However, because of the limited supply of broilers to the smaller integrated companies, broilers often originated from competitor farms. A summary of the management and production data on the four processing plants is shown in Table 1.

### 3.2. Comparison of Sampling Methods

**Figure 1:**
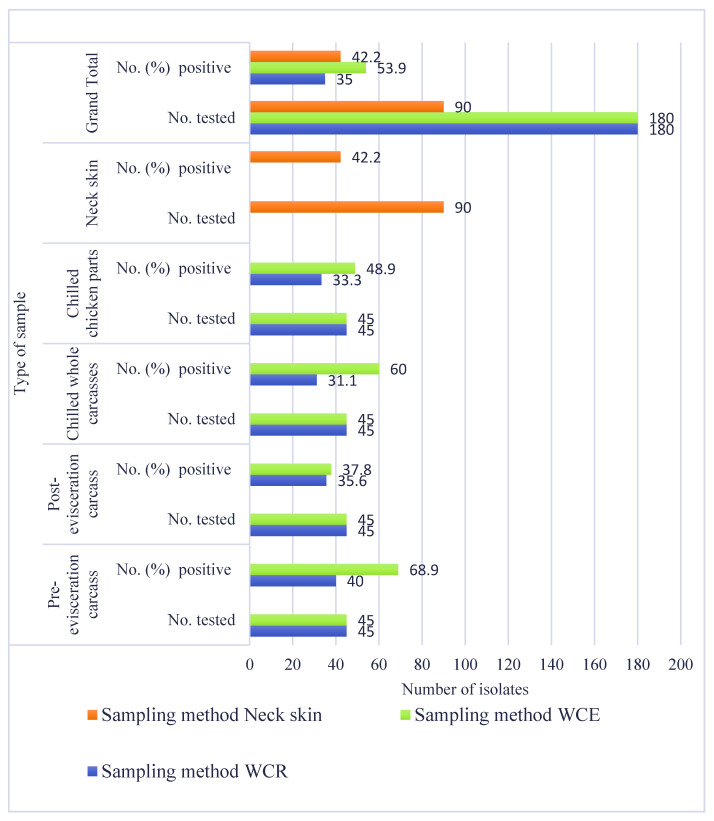
Recovery of *Salmonella* based on the method used.

*Salmonella* was isolated from 35.0%, 53.9%, and 42.2% of samples subjected to the WCR, WCE, and neck skin methods, respectively (*p* = 0.0013) (Figure 1). Significant differences in the frequency of isolation of *Salmonella* by sampling method were found in pre-evisceration carcasses (*p* < 0.001), post-evisceration carcasses (*p* < 0.001), and all samples (*p* < 0.001). Chilled whole carcasses subjected to the WCE method yielded a higher frequency of isolation (60%; 27/45) when compared to the WCR method (31.1%; 14/45) (*p* = 0.01). Selective enrichment in tetrathionate broth plated onto XLT-4 agar yielded the highest frequency of *Salmonella* positive samples among the three methods (*p* < 0.001). Overall, 8.9% (40/450), 29.8% (134/450), 1.8% (8/450), and 3.6% (16/450) of the samples were isolated on RVS/XLT-4, TT/XLT-4, RVS/BGA, and TT/BGA, respectively (*p* < 0.001).

### 3.3. Risk Factors Associated with Salmonella Contamination during Broiler Processing

The association of risk factors with the frequency of contamination of chickens processed is shown in Table 2. Of the 14 risk factors investigated, 10 (71.4%) were determined to be statistically significantly associated with the contamination with *Salmonella* during processing.

### 3.4. Multivariate Logistic Regression of Risk Factors for Isolation of Salmonella

Of the nine variables included in the initial logistic regression model, only the average number of contract farmers, the number of workers directly involved in the processing, and the waiting period were retained in the final model. Processing plants with more than 100 contract farms were significantly associated with increased odds of *Salmonella* isolation (OR = 8.5; χ2 = 16.968, *p* < 0.001) (Table 3). Similarly, plants where the waiting period between arrival and slaughter was more than 10 h were significantly associated with *Salmonella* isolation (OR = 2.9; χ2 = 4.072, *p* = 0.044). Plants, where there were more than 150 workers directly involved in processing, were included in the model but were not a significant predictor in the equation (*p* = 0.284). The Hosmer–Lemeshow test of goodness-of-fit test was not significant (χ2 = 0.00, *p* = 1), showing that the final logistic regression model fitted the data well.

### 3.5. Isolation from Different Broiler Processing Plants and Types of Samples

Overall, the isolation rate of *Salmonella* in carcasses sampled at broiler processing plants was 27.0% (107/396) (Table 4). Among all the samples collected during broiler processing, the isolation rate of *Salmonella* was highest in pre-evisceration carcasses (51.1%; 23/45) followed by chilled whole carcasses (44.4%; 20/45), chilled chicken parts (40.0%; 18/45), and post-evisceration carcasses (37.8%; 17/45) (Table 4). *Salmonella* was detected only in 2.2% (2/90) and 5.6% (2/36) cloacal swabs and immersion chiller water samples, respectively.

### 3.6. Serotypes of Salmonella Isolates

*S. enterica* serotype Enteritidis (20.8%; 15/72), Javiana (16.7%; 12/72), and Infantis (12.5%; 9/72) were the most prevalent among a total of 16 different serotypes isolated at broiler processing plants (Table 5). Serotypes Kentucky, Anatum, Schwarzengrund, and Albany were found in less than 10% of the isolates. Only one isolate each of serotypes Hindmarsh, Madjorio, Mbandaka, *S. enterica* subspecies Houtenae, Virchow, Weltevrden, Aberdeen, Alachua, and Ayinde were detected among all the isolates. Serotype Enteritidis was found primarily (14/15 samples: 93.3%) in chilled whole and chicken parts as well as neck skins.

### 3.7. Frequency of Resistance of Salmonella Isolates to Eight Antimicrobial Agents at Different Processing Plants

**Figure 2:**
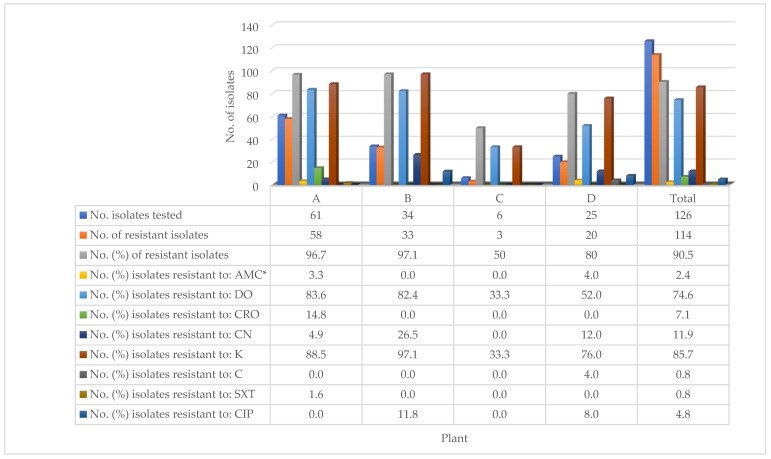
Antimicrobial resistance of *Salmonella* isolates isolated from four processing plants. * AMC, amoxicillin-clavulanic acid (30 µg); DO, doxycycline (30 µg); CRO, ceftriaxone (30 µg); CN, gentamicin (10 µg); K, kanamycin (30 µg); C, chloramphenicol (30 µg); SXT, sulfamethoxazole-trimethoprim (23.75 and 1.25 µg); CIP, ciprofloxacin (5 µg).

The prevalence of resistance to antimicrobial agents among *Salmonella* isolates tested was 90.5% (114/126) as resistance was exhibited to one or more of the eight antimicrobial agents tested (Figure 2). Overall, resistance was relatively high to K (85.7%) and DO (74.6%) but relatively low to SXT (0.8%), C (0.8%), and AMC (2.4%). The differences were statistically significant (*p* < 0.05). The overall prevalence of resistance to antimicrobial agents by *Salmonella* isolates was 96.7% (58/61), 97.1% (33/34), 50.0% (3/6), and 80.0% (20/25) at plant A, B, C, and D, respectively, and these differences were statistically significant (*p* < 0.05).

### 3.8. Frequency of Antimicrobial Resistance of Salmonella Isolates Based on the Type of Sample

The frequencies of resistance to antimicrobial agents (Table 6) were similar amongst the various types of samples, ranging from 86.2% to 100% in chilled chicken parts, post-evisceration carcasses, chilled whole carcasses, neck skins, pre-evisceration carcasses, chiller water, and cloacal swabs. The frequency of resistance to DO was significantly (*p* = 0.045) higher for isolates of *Salmonella* that originated from pre-evisceration carcasses (23/25, 92.0%) compared with isolates from other types of samples. The differences in the frequency of resistance were not statistically significant (*p* > 0.05) for *Salmonella* isolated from the other types of samples other than from chiller water samples.

### 3.9. Resistance of Salmonella Isolates Based on Serotype

Sixteen different serotypes of *Salmonella* were identified from the 72 isolates subjected to conventional serotyping. Serotypes Enteritidis and Javiana were the most prevalent serotypes with 60.0% (9/15) and 83.3% (10/12) exhibiting resistance to one or more agents, respectively (Table 7). All isolates (100.0%) belonging to serotypes Albany, Anatum, and Kentucky; 88.9% for Infantis, 83.3% for Javiana, and 83.3% for Schwarzengrund exhibited resistance to antimicrobial agents. Amongst the different serotypes, the differences in the resistance exhibited were only statistically significant to DO (*p* < 0.001).

### 3.10. Antimicrobial Resistance Patterns

A total of 14 (12.3%) of the 114 isolates of *Salmonella* exhibited multidrug resistance, i.e., resistance to antimicrobial agents belonging to three or more classes. Overall, a total of 12 different patterns were observed consisting of DO-K, which was the predominant pattern, with 54.4% isolates exhibiting the resistance pattern. Resistance to K alone was exhibited by 15 (13.2%) isolates, 12 (10.5%) isolates exhibited resistance to DO-CN-K, 8 (7.0%) exhibited resistance to DO-CRO-K, and 6 (5.3%) were resistant to DO alone. Other patterns observed ranged from 0.9% to 2.6% of resistant isolates.

## 4. Discussion

This is considered the first cross-sectional study conducted in the broiler processing plants in Trinidad and Tobago that documented the frequency of isolation of *Salmonella* along the processing lines. The study also characterized the isolates regarding their serotypes and antimicrobial resistance to currently used antimicrobial agents in the poultry industry. The food safety importance of the study cannot be underestimated because the four processing plants operational in the country supply the majority of local chickens and chicken products sold at supermarkets.

Of food safety concern, is the high level of contamination found in pre-packaged chilled whole carcasses (44.4%) and chilled chicken parts (40.0%) across the four processing plants. Salmonellosis has been reported in humans who consume inadequately cooked *Salmonella*-contaminated chicken meat [52,53]. Our findings agree with the prevalence of *Salmonella* found in chilled chicken carcasses in abattoirs elsewhere, where 48.0% [43], 45.2% [54], and 50.0% [55] were reported in the United States, China, and Brazil, respectively. These findings were higher than the 8.3% [56] and 3.75% [57] reported in Iran and the Czech Republic, respectively. It is interesting to note that the most recent study on the prevalence of *Salmonella* in chickens that originated from commercial processing plants in Trinidad was 8.3% [38]. The differences in the prevalence have been reported to be affected by the carriage of *Salmonella* during de-feathering [57], evisceration, and spray washing steps [58] as well as by contaminated chiller water [59].

The strategy used in our study which included the collection of samples from the time of reception of live chickens to the finished chilled chickens longitudinally, from the pre-evisceration samples to chilled carcasses during each visit provided evidence of statistically significant (*p* = 0.023) increased levels of contamination along the stages of processing. The differences in the frequencies of isolation of *Salmonella* in the samples between and within the four processing plants, could be due in part, to the different management, production, and risk factors at these plants. These findings were not surprising because other studies have reported progressive increases in the frequency of contamination with *Salmonella* during processing [60,61].

It is significant that the frequency of isolation of *Salmonella* from the cloacal swabs pre-slaughter across the four plants was 2.2% ranging from 0.0% to 10.0%. This is an indication that the prevalence of *Salmonella* was relatively low on the poultry farms from where the slaughtered birds originated. Our findings agree with the prevalence of *Salmonella* in cloacal swabs of broilers pre-slaughter reported in Trinidad and Tobago, 3.95% (3/76) [62]; Brazil, 7.0% (7/100) [20]; and Colombia, 12.5% (8/64) [63].

It was of epidemiological relevance to have detected that 71.4% of the 14 risk factors investigated demonstrated statistically significant association with the contamination of chicken carcasses during processing at the plants. Significantly higher frequencies of isolation of *Salmonella* were detected among the following factors including medium-sized plants, use of more than 100 contract farmers, employment of less than 150 workers directly involved in processing, the average mortality rate of over 0.5% in broilers on arrival at the plant, i.e., dead on arrival, the use of pre-chillers, and the use of sanitizers in chiller water, used sanitizers for general cleaning of plants, among other factors. Many of these risk factors have been documented to be associated with the isolation of *Salmonella* in processing plants by others [15,64,65,66,67]. Standardized sanitation protocols with surveillance to monitor the efficacy and the development of resistance is suggested. In addition, frequent training programs for processing plant workers and farmers to educate them on the current best-practices will be beneficial in reducing cross-contamination along the continuum. Interestingly, further regression analyses and the odds ratio (OR) revealed that *Salmonella* was 4.4 more likely (95% CI: 2.68–7.34) to be isolated from chickens in plants that received birds from more than 100 farmers. This risk could be attributed to the increased possibility of slaughtering broilers from *Salmonella*-infected farms. Similarly, it was detected that plants that allowed the slaughter of chickens from batches with mortality rates of over 0.5% on arrival at the plants were 2.3 times more likely (95% CI: 1.45–3.74) to lead to the isolation of *Salmonella* from chickens at those plants. Although the specific pathogens responsible for deaths experienced during transportation to the plant were not known, the possibility exists that *Salmonella* may be involved. The contamination of feathers of chickens from direct contact with feces of infected broilers shedding *Salmonella* and exposure to the pathogen in the transport vehicle on its way to the plant has been documented [68,69]. Similarly, the risk of contamination of chickens increased considerably by 4.4 times (95% CI: 2.68–7.34) in plants that permitted the slaughter of sick birds, albeit being processed last instead of being rejected at farms. The possibility of seeding the plant environment with pathogens, including *Salmonella*, is pertinent, if the cleaning of the plant is inadequate. *Salmonella* was isolated at a significantly higher frequency in plants that used chlorine (29.0%) than those that used hot water (11.4%). This is because *Salmonella* has been reported to develop resistance to sanitizers [70,71,72]. Additionally, our study noted that plants that used pre-chillers but did not add chemical agents were found to be 1.7 times more likely (95% CI: 0.94–3.02) to result in the recovery of *Salmonella*. The proper use of chillers and sanitizers in processing plants can therefore not be ignored [73,74].

In our study, the WCE method yielded a statistically significant higher (53.9%) frequency of isolation of *Salmonella* than either the WCR (35.0%) or the NS (42.2.%) methods, making it the most sensitive method for *Salmonella* detection as reported by others [43,75]. Berrang et al. [36] attributed this increased sensitivity to the ability of the WCE method to facilitate the proliferation of *Salmonella* in low quantities or those firmly attached to the skin of the chicken. However, the challenges associated with WCE method, particularly the considerably larger incubator space requirement compared with the use of WCR and NS methods, cannot be disregarded thereby making it an impractical method for routine surveillance testing but applicable as a research tool. It has been reported that the types of samples and the methods of enrichment affect their sensitivities to detect *Salmonella* in chickens [76,77].

The predominant serotypes of *Salmonella* isolated were Enteritidis, Javiana, and Infantis. These serotypes have similarly been isolated from chicken-associated samples in the country, such as chickens sampled from supermarkets that originated from broiler processing plants and outlets of cottage poultry processors [38] and chicken layers [78]. In the current study, it was found that the serotypes were detected at different frequencies from the types of samples tested in the processing plants, a finding that agrees with published reports [79,80]. Of food safety and public health, the significance is the fact that some of these predominant serotypes were determined in the Caribbean Public Health Agency (CARPHA) State of Public Health report [81], to be amongst the top 15 human *Salmonella* serotypes detected in the region. Similarly, the predominant serotypes in our study were also reported to be the most commonly *Salmonella* serotypes associated with human salmonellosis in Trinidad and Tobago between 2005–2012 [81]. It cannot be underestimated that serotype Enteritidis has globally been associated with poultry meat and eggs, and responsible for human cases and epidemics of salmonellosis [82,83].

The high prevalence of resistance (90.5%) to antimicrobial agents by the 126 isolates of *Salmonella* recovered from the four processing plants, has both zoonotic and therapeutic implications. It is important to have detected that the high prevalence of resistance was exhibited to antimicrobial agents routinely used in the poultry industry in the country. It has been reported that zoonotic spread of *Salmonella* to workers at the commercial processing plants may occur [84,85] and as well as therapeutic failure in consumers of improperly cooked chickens contaminated by antimicrobial resistant-*Salmonella* [80]. Similarly, a high prevalence of resistance to antimicrobial agents (100.0%) has been reported in chilled chickens from supermarkets and cottage poultry processors [39]. Although the current study was not farm-based, the prevalence of resistant *Salmonella* in chickens processed at the plants may be indicative of the level of resistance of *Salmonella* on the contract farms from where they originated. It has been documented that the misuse or over-use of antimicrobial agents by farmers may result in the development of resistance to antimicrobial agents [86]. This is a common practice particularly in developing countries, including Trinidad and Tobago, where although laws governing the type and use of antimicrobial agents for prophylaxis, growth promotion, and therapy exist, prevailing challenges limit or prevent their enforcement [87,88].

With regard to the eight antimicrobial agents tested, it was important that the overall prevalence of resistance was comparatively low (0.8–11.9%) to six (amoxicillin-clavulanic acid, ceftriaxone, gentamicin, chloramphenicol, sulphamethoxazole–trimethoprim, and ciprofloxacin) of the antimicrobial agents, while significantly higher prevalence was exhibited to doxycycline (74.6%) and kanamycin (85.7%). Furthermore, the study found that the prevalence of resistance to the antimicrobial agents varied significantly across the processing plants from where the *Salmonella* isolates originated. These findings reflect the differences in the types and the frequency of use of antimicrobial agents on the contract farms that supplied live broilers to the plants. The high prevalence of resistance exhibited to doxycycline and kanamycin has been documented in chickens in the country [39]. The detection of a high prevalence of resistance (60.0% to 88.9%) among the top three detected serotypes (Enteritidis, Javiana, and Infantis) may also be therapeutic significance to infected broilers or humans. Differences in the prevalence of resistance to antimicrobial agents by *Salmonella* have been reported to vary among serotypes of *Salmonella* from chickens by others [71,89]. Therefore, there is a need to monitor the use of the two antimicrobial agents on broiler farms in the country.

It is concluded that the high prevalence of *Salmonella* (27.0%) including antimicrobial-resistant strains (90.5%), along with the predominance of three serotypes (Enteritidis, Javiana, and Infantis) among the isolates has implications for human salmonellosis in the country. The relative risk of salmonellosis posed by consumption of under-cooked *Salmonella*-contaminated chicken meat from these plants needs to be emphasized. The fact that 10 of the 14 risk factors investigated were statistically significantly associated with the contamination of chicken in the processing lines along with the odds ratio (OR) generated provides critical control points where interventions may be successfully applied. Our study reveals that the WCE method, which is not used for routine surveillance of *Salmonella* in chickens, demonstrated its significantly higher sensitivity when compared with either the WCR or NS methods, a finding that may be indicative of the potential under-reporting of the prevalence of antimicrobial resistant *Salmonella* in chickens in the country. The high prevalence of antimicrobial resistance exhibited by *Salmonella* isolates in this study poses both zoonotic and therapeutic implications to humans exposed to infected chickens. It is imperative to control the use of antimicrobial agents on poultry farms to reduce the development of antimicrobial resistance among *Salmonella*.

## Figures and Tables

**Table 1:** Management and production data from four broiler processing plants in Trinidad.

Parameter	Processing Plant:			
	Plant A	Plant B	Plant C	Plant D
Total installed capacity of the processing plant (birds/week)	160,000	250,000	<100,000	100,000
Average number of broilers processed daily	32,000	50,000	15,000	20,000
Number of days operational weekly	5	5	4	5
Average number of contract farmers used	210	32	32	100
Number of workers directly involved in Processing ^a^	150	400	75	150
Number of workers indirectly involved in Processing ^b^	100	1000	1000	75
Waiting period (h) between arrival of birds at plant and slaughter	2–6	0.5–3	1–3	12
Average mortalities (%) or broilers dead on arrival at plant	0.7	0.02	0.94	0.50
Disposal of solid waste (fecal materials) from broilers	Rendered ^c^	External Company	Rendered	Rendered
Disposal of waste-water	River	Settling ponds	Settling ponds	Settling ponds
Treatment of water at the plant ^d^	No	Yes	No	No

^a^ Workers who have contact with the birds/carcass at one point during processing; ^b^ Workers involved in the management of the plant but not having contact with the birds/carcass during processing; ^c^ Rendering (in-house) to convert animal tissue waste to useable by-product meal; ^d^ All plants utilized municipal water supply as their source.

**Table 2:** Risk factors associated with *Salmonella* contamination of carcasses.

Risk Factor	Total No. Samples Tested	Total No. (%) Positive for *Salmonella*	*p*-Value	Odds Ratio	CI (95%)
Size of plant ^a^			*p* < 0.001		
Small	44	5 (11.4)		Ref	
Medium	176	70 (39.8)		5.1	1.94–13.71
Large	176	32 (18.2)		1.7	0.63–4.74
Average number of contract farmers			*p* < 0.001		
≤100 farmers	308	61 (19.8)		Ref	
>100 farmers	88	46 (52.3)		4.43	2.68–7.34
Number of workers directly involved in processing operation			*p* = 0.001		
≤150 workers	220	75 (34.1)		Ref	
>150 workers	176	32 (18.2)		0.43	0.27–0.70
Average waiting period from arrival at plant to processing			*p* = 0.95		
≤10 h	308	83 (26.9)		Ref	
>10 h	88	24 (27.3)		1.01	0.60–1.73
Average mortality rate (%) of birds on arrival at plant			*p* = 0.001		
<0.50	176	32 (18.2)		Ref	
≥0.50	220	75 (34.1)		2.32	1.45–3.74
Handling of sick/diseased birds			*p* < 0.001		
Rejected at farm	308	61 (19.8)		Ref	
Processed last	88	46 (52.3)		4.43	2.68–7.34
Use of pre-chiller			*p* = 0.001		
Yes	220	75 (34.1)		Ref	
No	176	32 (18.2)		2.32	1.45–3.74
Agents used in pre-chiller ^b^			*p* = 0.11		
Citric acid + chlorine	88	24 (27.3)		Ref	
No agents added	132	51 (38.6)		1.67	0.94–3.02
Temperature of pre-chiller ^b^			*p* < 0.001		
Room temperature	88	46 (52.3)		Ref	
10 °C	44	5 (11.4)		0.11	0.04–0.33
20 °C	88	24 (27.3)		0.34	0.18–0.64
Agents used in chiller			*p* = 0.01		
Chlorine	352	102 (29.0)		3.18	1.22–8.30
No agents added ^c^	44	5 (11.4)		Ref	
Concentration of chlorine used in chiller ^c^			*p* = 0.79		
20 ppm	88	24 (27.3)		Ref	
21–50 ppm	264	78 (29.5)		1.11	0.65–1.92
Temperature of chiller			*p* = 0.14		
<1 °C	132	29 (22.0)		Ref	
1–4 °C	264	78 (29.5)		1.49	0.91–2.43
Agents used for general cleaning of plant during processing			*p* = 0.01		
Sanitizer	352	102 (29.0)		Ref	
Hot water only	44	5 (11.4)		0.31	0.12–0.82
Worker segregation ^d^			*p* = 0.01		
Yes	352	102 (29.0)		3.18	1.22–8.30
No	44	5 (11.4)		Ref	

^a^ Based on weekly throughput, small <100,000 birds; medium 101,000–160,000 birds; large >161,000 birds. ^b^ Only 3 plants use pre-chillers. ^c^ Only 3 plants add additional chlorine to chiller water. Chlorine concentration ranged from 1–5 ppm in the municipal water supply. ^d^ Colour coding of workers was done to limit movement of workers to prevent cross contamination of dirty and clean work areas.

**Table 3:** Results of a multivariate logistic regression of risk factors for *Salmonella* isolation from carcasses sampled at broiler processing plants in Trinidad.

Variable	Coef.	Standard Error ^a^	Chi-Square	*p*-Value	Odds Ratio	95.0% CI	
						Lower	Upper
**>100 versus ≤100 farmers**	2.145	0.521	16.968	<0.001	8.543	3.078	23.707
**>150 versus ≤150 workers**	0.55	0.514	1.147	0.284	1.733	0.633	4.744
**>10 h versus ≤10 h waiting period**	1.073	0.532	4.072	0.044	2.925	1.031	8.296
**Constant**	−2.054	0.475	18.7	<0.001	0.128		

^a^ Standard error of the coefficient.

**Table 4:** Frequency of isolation of *Salmonella* by type of samples tested at each plant.

Stage in Processing	Type of Sample Collected	Plant A		Plant B		Plant C		Plant D		Total No. Tested	Total No. (%) Positive for *Salmonella*
		No. of Samples Tested	No. (%) Positive	No. of Samples Tested	No. (%) Positive	No. of Samples Tested	No. (%) Positive	No. of Samples Tested	No. (%) Positive		
Pre-evisceration	Cloacal swab	20	2 (10.0)	40	0 (0.0)	10	0 (0.0)	20	0 (0.0)	90	2 (2.2)
	De-feathered carcass	10	7 (70.0)	20	9 (45.0)	5	1 (20.0)	10	6 (60.0)	45	23 (51.1)
	*p*-value		0.002		<0.001		0.333		0.0004		<0.001
	Subtotal	30	9 (30.0)	60	9 (30.0)	15	1 (6.7)	30	6 (20.0)	135	25 (18.5)
Post-evisceration	Eviscerated carcass	10	7 (70.0)	20	5 (25.0)	5	0 (0.0)	10	5 (50.0)	45	17 (37.8)
	Neck skin	20	14 (70.0)	40	9 (22.5)	10	0 (0.0)	20	2 (10.0)	90	25 (27.8)
	*p*-value		0.656		0.535		NA		0.026		0.972
	Subtotal	30	21 (70.0)	60	14 (23.3)	15	0 (0.0)	30	7 (23.3)	135	42 (31.1)
Chiller water and carcasses	Chilled water	8	0 (0.0)	16	2 (12.5)	4	0 (0.0)	8	0 (0.0)	36	2 (5.6)
	Chilled-whole carcass	10	7 (70.0)	20	5 (25.0)	5	3 (60.0)	10	5 (50.0)	45	20 (44.4)
	Chilled-parts	10	9 (90.0)	20	2 (10.0)	5	1 (20.0)	10	6 (60.0)	45	18 (40.0)
	*p*-value		0.0004		0.391		0.123		0.024		0.0003
	Subtotal	28	16 (57.1)	56	9 (16.1)	14	4 (28.6)	28	11 (39.3)	126	40 (31.7)
Total		88	46 (52.3)	176	32 (18.2)	44	5 (11.4)	88	24 (27.3)	396	107 (27.0)
	*p*-value										<0.001
Pre-evisceration		30	9 (30.0)	60	9 (30.0)	15	1 (6.7)	30	6 (20.0)	135	25 (18.5)
Post-evisceration		30	21 (70.0)	60	14 (23.3)	15	0 (0.0)	30	7 (23.3)	135	42 (31.1)
Chiller water and carcasses		28	16 (57.1)	56	9 (16.1)	14	4 (28.6)	28	11 (39.3)	126	40 (31.7)
	*p*-value		0.007		0.439		0.041		0.215		0.023

**Table 5:** *Salmonella* serotypes isolated from different types of samples.

Stage of Processing	No. of Samples Positive for *Salmonella*	No. (%) ^a^ of Isolates Serotyped	Serotypes (No., %)
Cloacal swabs	2	0 (0.0)	Not applicable
Pre-evisceration carcass	23	2 (8.7)	Weltevreden (1, 50.0)
			Enteritidis (1, 50.0)
Post-evisceration carcass	17	5 (29.4)	Javiana (3, 60.0)
			Virchow (1, 20.0)
			Infantis (1, 20.0)
Neck skins	25	25 (100.0)	Javiana (7, 28.0)
			Schwarzengrund (5, 20.0)
			Albany (4, 16.0)
			Anatum (3, 12.0)
			Infantis (2, 8.0)
			Group C2 ^b^ (2, 8.0)
			Madjorio (1, 4.0)
			Enteritidis (1, 4.0)
Chiller water	2	2 (100.0)	*Salmonella* spp. (1, 50.0)
			subspecies Houtenae IV (1, 50.0)
Chilled whole carcass	20	20 (100.0)	Enteritidis (7, 35.0)
			Infantis (4, 20.0)
			Anatum (2, 10.0)
			Albany (1, 5.0)
			Mbandaka (1, 5.0)
			Schwarzengrund (1, 5.0)
			Aberdeen (1, 5.0)
			Javiana (1, 5.0)
			Kentucky (1, 5.0)
			Ayinde (1, 5.0)
Chilled chicken parts	18	18 (100.0)	Enteritidis (6, 33.3)
			Kentucky (6, 33.3)
			Infantis (2, 11.1)
			Hindmarsh (1, 5.6)
			Javiana (1, 5.6)
			Anatum (1, 5.6)
			Alachua (1, 5.6)
Total	107	72 (67.3)	

^a^ Of the number of randomly selected *Salmonella* serotypes from each source; ^b^ Serogroup (Group C2) could not be determined to the serotype level.

**Table 6:** Antimicrobial resistance of *Salmonella* isolated from various stages of processing.

Stage in Processing	Type of Sample Collected	No. of Isolates Tested	No. (%) of Isolates Resistant ^a^	No. (%) Resistant to ^b^:							
				AMC	DO	CRO	CN	K	C	SXT	CIP
Pre-evisceration	Cloacal swab	3	3 (100.0)	0 (0.0)	1 (33.3)	0 (0.0)	1 (33.3)	3 (100.0)	0 (0.0)	0 (0.0)	0 (0.0)
	Defeathered carcass	25	24 (96.0)	0 (0.0)	23 (92.0)	1 (4.0)	2 (8.0)	23 (92.0)	0 (0.0)	0 (0.0)	0 (0.0)
	*p*-value		1	NA	0.045	1	0.298	1	NA	NA	NA
	Subtotal	28	27 (96.4)	0 (0.0)	24 (85.7)	1 (3.6)	3 (10.7)	26 (92.9)	0 (0.0)	0 (0.0)	0 (0.0)
Post-evisceration	Eviscerated Carcass	17	15 (88.2)	0 (0.0)	12 (70.6)	0 (0.0)	2 (11.8)	14 (82.4)	0 (0.0)	0 (0.0)	0 (0.0)
	Neck skin	25	23 (92.0)	0 (0.0)	18 (72.0)	2 (8.0)	6 (24.0)	21 (84.0)	0 (0.0)	0 (0.0)	3 (12.0)
	*p*-value		1	NA	1	0.506	0.439	1	NA	NA	0.260
	Subtotal	42	38 (90.5)	0 (0.0)	30 (71.4)	2 (4.8)	8 (19.0)	35 (83.3)	0 (0.0)	0 (0.0)	3 (7.1)
Chiller water and carcasses	Chiller water	2	2 (100.0)	0 (0.0)	0 (0.0)	0 (0.0)	0 (0.0)	2 (100.0)	0 (0.0)	0 (0.0)	1 (50.0)
	Chilled-whole carcass	29	25 (86.2)	0 (0.0)	20 (69.0)	4 (13.8)	4 (13.8)	24 (82.8)	0 (0.0)	0 (0.0)	2 (6.9)
	Chilled-parts	25	22 (88.0)	3 (12.0)	20 (80.0)	2 (8.0)	0 (0.0)	21 (84.0)	1 (4.0)	1 (4.0)	0 (0.0)
	*p*-value		0.846	0.140	0.050	0.698	0.135	0.814	0.532	0.532	0.009
	Subtotal	56	49 (87.5)	3 (5.4)	40 (71.4)	6 (10.7)	4 (7.1)	47 (83.9)	1 (1.8)	1 (1.8)	3 (5.4)
Total		126^c^	114 (90.5)	3 (2.4)	94 (74.6)	9 (7.1)	15 (11.9)	108 (85.7)	1 (0.8)	1 (0.8)	6 (4.8)
Pre-evisceration		28	27 (96.4)	0 (0.0)	24 (85.7)	1 (3.6)	3 (10.7)	26 (92.9)	0 (0.0)	0 (0.0)	0 (0.0)
Post-evisceration		42	38 (90.5)	0 (0.0)	30 (71.4)	2 (4.8)	8 (19.0)	35 (83.3)	0 (0.0)	0 (0.0)	3 (7.1)
Chiller water and carcasses		56	49 (87.5)	3 (5.4)	40 (71.4)	6 (10.7)	4 (7.1)	47 (83.9)	1 (1.8)	1 (1.8)	3 (5.4)
	*p*-value		0.422	0.147	0.310	0.373	0.193	0.471	0.533	0.533	0.374

^a^ Resistance to one or more agents tested. ^b^ AMC, amoxicillin-clavulanic acid (30 µg); DO, doxycycline (30 µg); CRO, ceftriaxone (30 µg); CN, gentamicin (10 µg); K, kanamycin (30 µg); C, chloramphenicol (30 µg); SXT, sulfamethoxazole-trimethoprim (23.75 and 1.25 µg); CIP, ciprofloxacin (5 µg). ^c^ A total of 126 isolates may have included duplicates of isolates obtained from TT/XLT-4, TT/BGA, RVS/XLT-4, and RVS/BGA media, solely of phenotypes. NA: Not applicable.

**Table 7:** Resistance exhibited by different serotypes isolated at four processing plants.

Serotype ^a^	No. of Isolates Tested	No. (%) of Isolates Resistant ^b^	No. (%) Isolates Resistant to ^c^:							
			AMC	DO	CRO	CN	K	C	SXT	CIP
Albany	5	5 (100.0)	0 (0.0)	5 (100.0)	2 (40.0)	0 (0.0)	4 (80.0)	0 (0.0)	0 (0.0)	0 (0.0)
Anatum	6	6 (100.0)	0 (0.0)	6 (100.0)	2 (33.3)	0 (0.0)	4 (66.7)	0 (0.0)	0 (0.0)	0 (0.0)
Enteritidis	15	9 (60.0)	1 (6.7)	1 (6.7)	0 (0.0)	0 (0.0)	9 (60.0)	1 (6.7)	0 (0.0)	2 (13.3)
Infantis	9	8 (88.9)	0 (0.0)	6 (66.7)	0 (0.0)	2 (22.2)	8 (88.9)	0 (0.0)	0 (0.0)	0 (0.0)
Javiana	12	10 (83.3)	0 (0.0)	10 (83.3)	0 (0.0)	4 (33.3)	10 (83.3)	0 (0.0)	0 (0.0)	3 (25.0)
Kentucky	7	7 (100.0)	2 (28.6)	7 (100.0)	1 (14.3)	0 (0.0)	6 (85.7)	0 (0.0)	1 (14.3)	0 (0.0)
Schwarzengrund	6	5 (83.3)	0 (0.0)	4 (66.7)	0 (0.0)	2 (33.3)	5 (83.3)	0 (0.0)	0 (0.0)	0 (0.0)
*p*-value			0.813	<0.001	0.252	0.238	0.745	1	0.996	0.192
Total	60	50 (83.3)	3 (5.0)	39 (65.0)	5 (8.3)	8 (13.3)	46 (76.7)	1 (1.7)	1 (1.7)	5 (8.3)

^a^ In addition, 2 (100.0%) of 2 Group C2 isolates exhibited resistance to one or more of the eight antimicrobial agents tested; 1 (100.0%) of 1 of the following serotypes Aberdeen, Alachua, Ayinde, Hindmarsh, Madjorio, Mbandaka, *Salmonella* sp. (untypable), S. Houtenae, Virchow, and Weltevreden were resistant, i.e., a total of 12 isolates. ^b^ Exhibited resistance to one or more antimicrobial agents. ^c^ AMC, amoxicillin-clavulanic acid (30 µg); DO, doxycycline (30 µg); CRO, ceftriaxone (30 µg); CN, gentamicin (10 µg); K, kanamycin (30 µg); C, chloramphenicol (30 µg); SXT, sulfamethoxazole-trimethoprim (23.75 and 1.25 µg); CIP, ciprofloxacin (5 µg).

## Data Availability

All the data are contained within the article and the Supplementary Materials.

## Supplementary Materials

Available online at https://www.mdpi.com/article/10.3390/microorganisms9051048/s1: S1: Flow chart of activities that take place at the four processing plants, S2: Broiler processing plant questionnaire.
