# Supplementary material for: Characterization of Salmonella Isolates Recovered from Stages of the Processing Lines at Four Broiler Processing Plants in Trinidad and Tobago
Source: Microorganisms. 2021 May 13;9(5):1048. doi: 10.3390/microorganisms9051048 (PMC8152471; doi:10.3390/microorganisms9051048)
Supplement: Supplementary file 1 [file microorganisms-09-01048-s001.zip › microorganisms-1200148-supplementary.pdf]

Supplemental data. S1 A-D

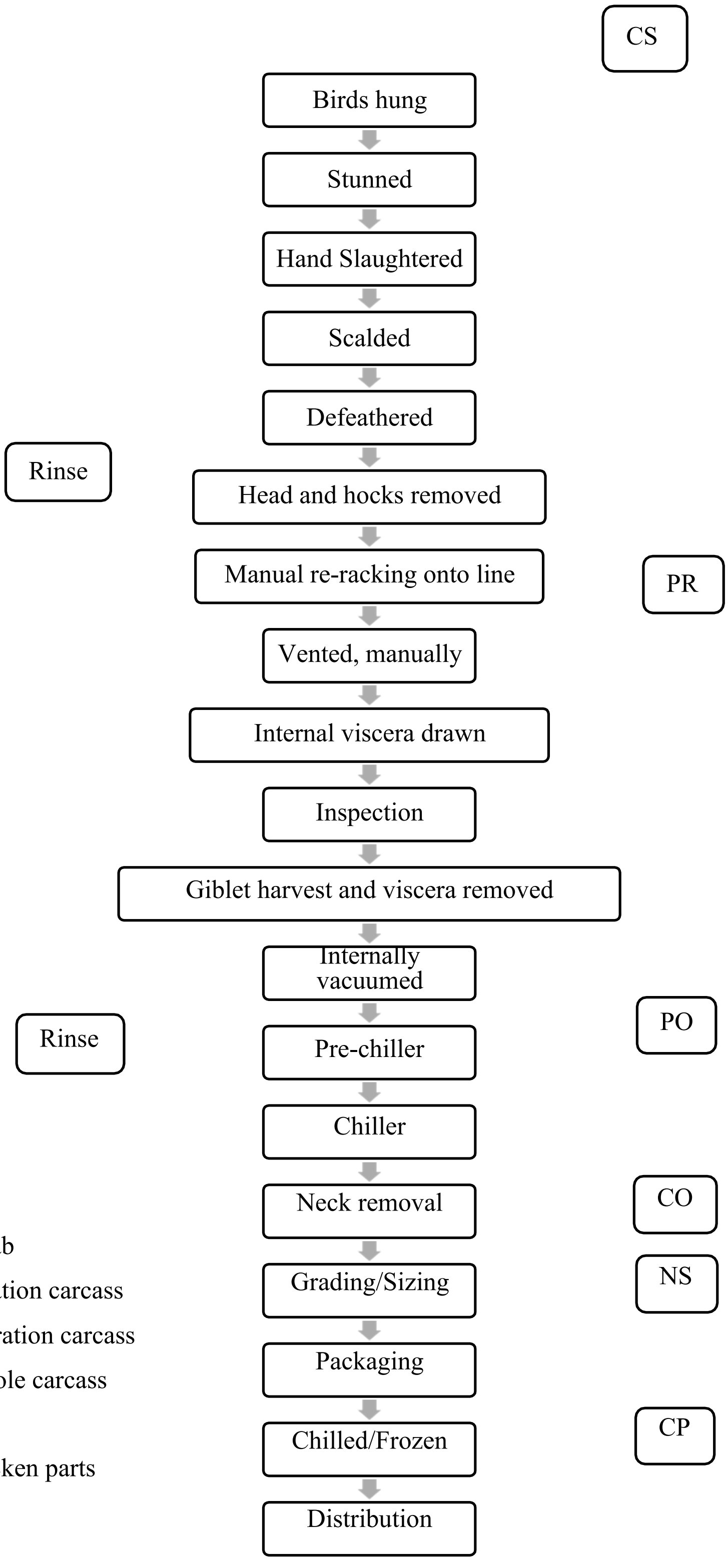

Key:

CS- Cloacal swab

PR- pre-evisceration carcass

PO- post-evisceration carcass

CO- Chilled whole carcass

NS- Neck skin

CP- Chilled chicken parts

Supplementary data S1 A. Flow chart showing processing at Plant A

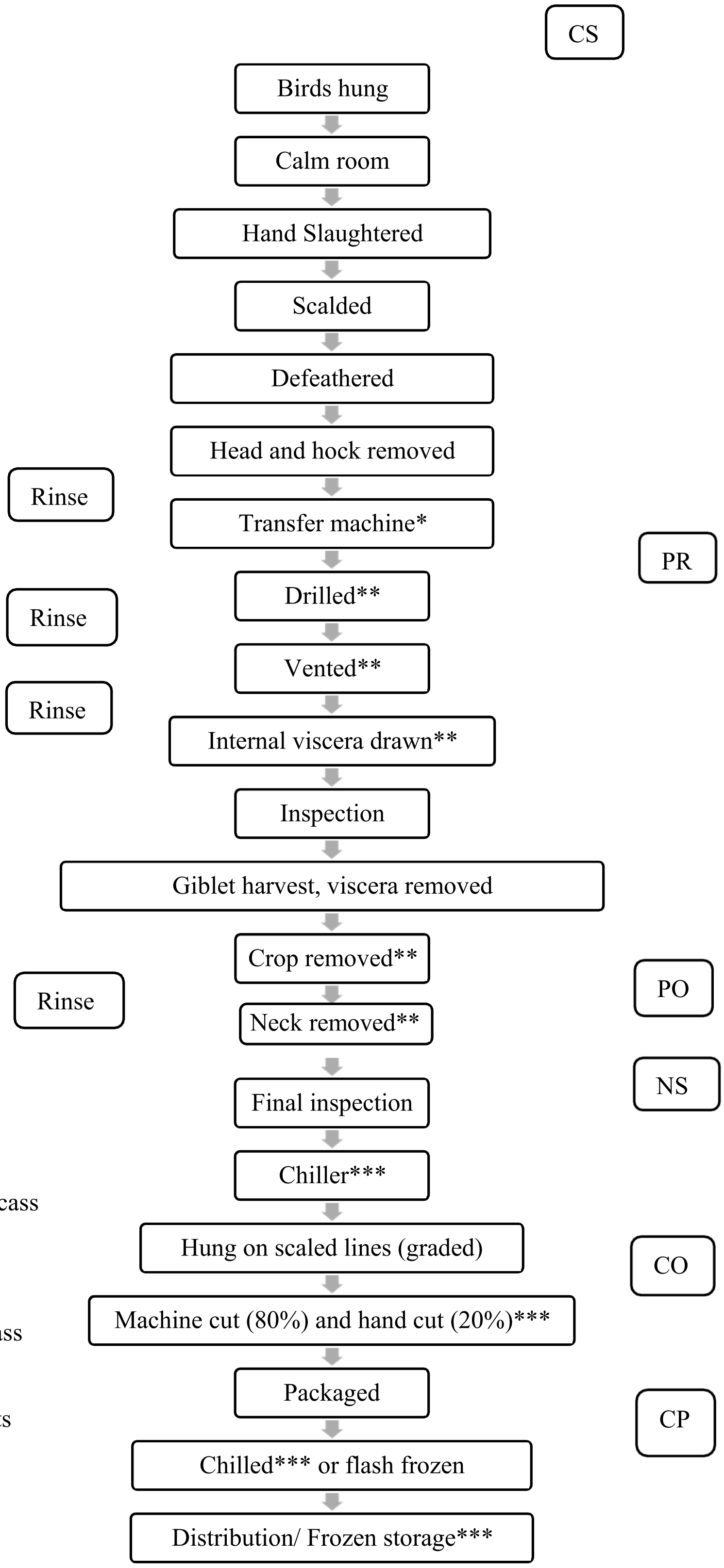

Key:

CS- Cloacal swab

PR- pre-evisceration carcass

PO- post-evisceration carcass

CO- Chilled whole carcass

NS- Neck skin

CP- Chilled chicken parts

Supplementary data S1 B. Flow chart showing processing at Plant B.

*Slaughter line transferred to evisceration line

**Automated/machine controlled process

***Critical control point

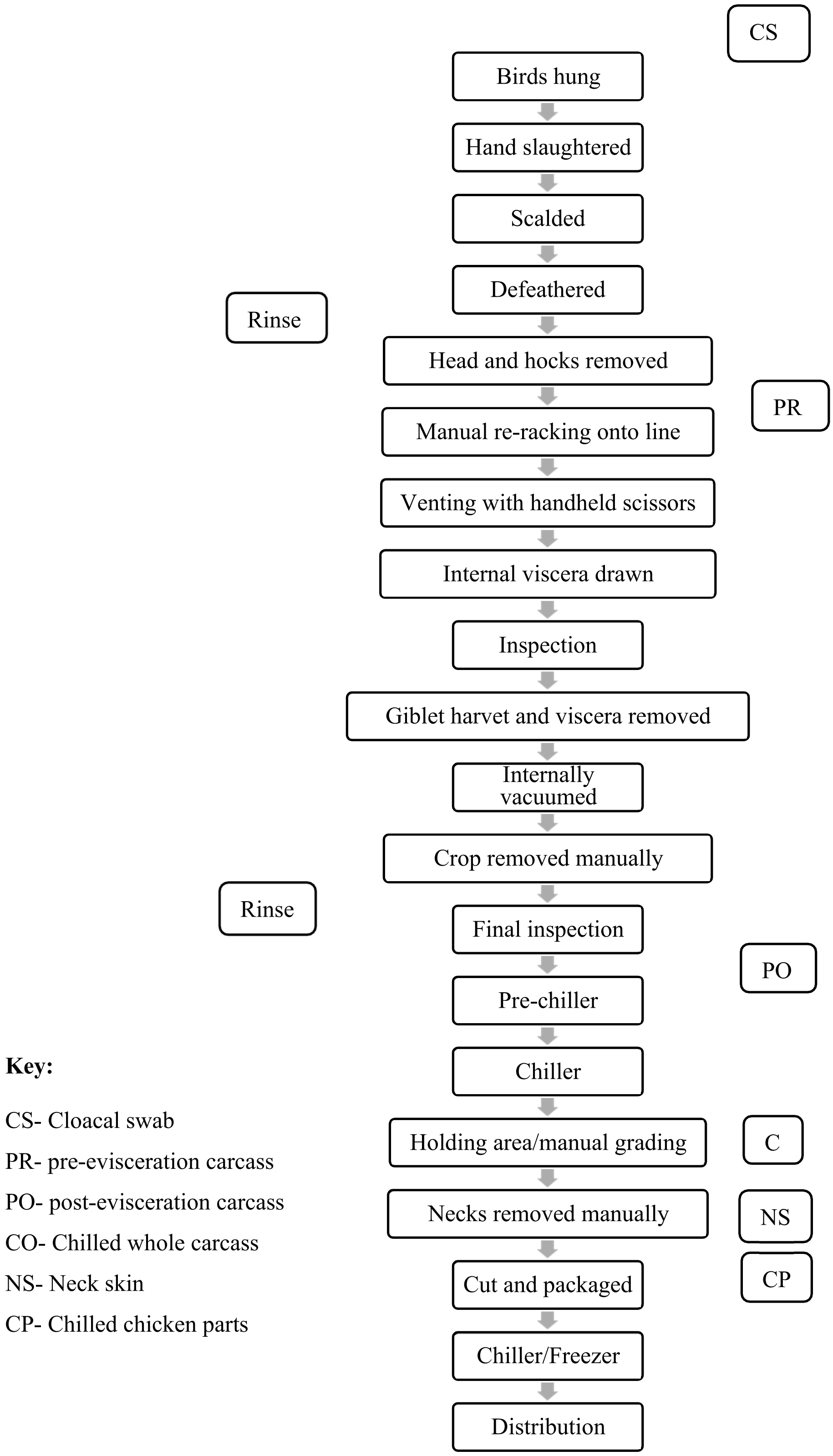

Supplementary data S1 C. Flow chart of processing at Plant C

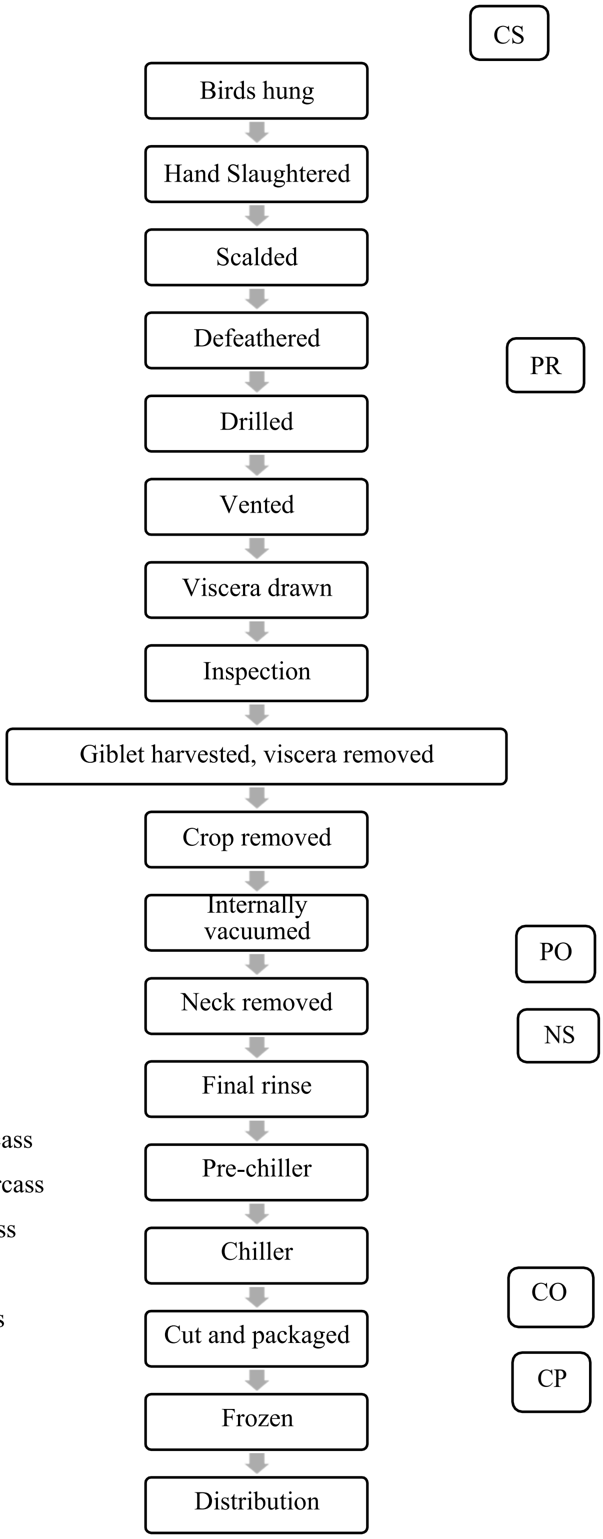

Key:

CS- Cloacal swab

PR- pre-evisceration carcass

PO- post-evisceration carcass

CO- Chilled whole carcass

NS- Neck skin

CP- Chilled chicken parts

Supplementary data S1 D. Flow chart showing processing at Plant D

Supplementary data S2. Questionnaire administered to processing plants managers

THE UNIVERSITY OF THE WEST INDIES
FACULTY OF MEDICAL SCIENCES
SCHOOL OF VETERINARY MEDICINE

QUESTIONNAIRE

Prevalence and characteristics of *Salmonella* spp. isolated from broiler processing plants in Trinidad

SECTION A: Plant Information

A1. Date: __________ A2. Code: _________

A3. GPS: ___________________________
____

A4. Name of Abattoir

A5. Contact Person

A6. Address of Abattoir

A7. Telephone Number A8. Email Address

A9. How many birds are slaughtered at this processing plant for the week?
1.☐500-1000 birds 2.☐1001-5000 birds 3.☐5001-10,000 birds
4.☐ 10,001-15,000 birds 5.☐ 15,001-20,000birds 6.☐20,001-25,000 birds
7.☐25,001-30,000 birds 8.☐ > 30,001 birds 9.☐ Other: ___________

A10. a) How many birds are expected to be slaughtered today (day of sampling)
1.☐500-1000 birds 2.☐1001-5000 birds 3.☐5001-10,000 birds
4.☐ 10,001-15,000 birds 5.☐ 15,001-20,000birds 6.☐20,001-25,000 birds
7.☐25,001-30,000 birds 8.☐ > 30,001 birds

A10. b) How many farms are bringing broilers to process today? ___________________

A11. How many days/week do you process broilers?
1.☐ 1-2 days/week 2.☐ 3-4 days/week 3.☐ 5 days/week
4.☐ 7 days/week

A12. How long has this plant been in operation?
____

A13. Do you export the final product? 1☐ Yes 2☐ No
A14. If Yes,

1. Country exported too	2. Product/s exported (whole or parts	3. Frequency of exports/shipment
a.		
b.		
c.		
d.		

e.		
f.		

SECTION B: Operations

B1. List the names of farms supplying broilers to the plant and quantity supplied today (Tick farm where cloacal swabs taken from)

Visit #	Name of Broiler Farm	Location of farm	Quantity supplied today
1)			
2)			
3)			
4)			

B2. How many workers are directly involved in the processing operation i.e. handling of the carcass in all shifts?

1.☐ 1-25　2. ☐ 26-50　3. ☐ 51-75　4. ☐ 75-100

5. ☐ 101-125　6. ☐ 126-150　7. ☐ 151-175　8. ☐ 176-200

9. ☐ 201-225　10.☐ 226 or over

B3. How many workers are indirectly (overhead) involved in the processing operation?

1. ☐ 1-25　2. ☐ 26-50　3. ☐ 51-75　4. ☐ 75-100

5. ☐ 101-125　6. ☐ 126-150　7. ☐ 151-175　8. ☐ 176-200

9. ☐ 201-225　10.☐ 226 or over

B4. How long is the waiting period between arrival of birds and slaughter?

1. ☐ 15-30 mins　2. ☐ 30-45 mins　3. ☐ 45-60 mins

4. ☐ > 60 min (1 h)

B5. Are there any measures in place to reduce stress in birds on arrival?

1. ☐ Covered area　2. ☐ High ceiling　3. ☐ Fans　4. ☐ Water spray

5. ☐ Others (mention)____

B6. What is the average mortality/death rate (%) of broilers on arrival at plant i.e. Of the total number of birds brought to the plant daily, how many arrived dead?

____

B7. If birds from a farm appear to be diseased, how does this affect its processing?

1. ☐ Proceed as normal　3. ☐ Process as last batch for the day

2. ☐ Reject　4. ☐Other (specify):_______________________

____

B8. Please list key steps in the operation.

Location	Temperature	Time spent here	Outflow rate/hr	Agents/chemicals added	Concentrations maintained
Pre- chiller					
Chiller					

SECTION C: Sanitary Practices/Protocols employed

C1. How do you dispose of the faecal material?
____
____
____

C2. How do you dispose of the offal?
____
____
____

C3. How do you dispose of the liquid/effluent waste?
____
____

C4. How do you dispose of dead carcasses pre-slaughter?
____
____
____

C5. Is the liquid waste treated before release into public waterways? 1. ☐ Yes 2. ☐ No

C6. If yes, state chemicals/processed used.

1	
2	
3	
4	
5	
6	

C7. How often is quality control (QC)- bacterial contamination analysis performed?

1. ☐ Everyday 2.☐ Every other day 3.☐ 3-4 times a week
4. .☐ Others (specify)____

C8. List the types of samples tested for QC?

1. ____________________________
2. ____________________________
3. ____________________________
4. ____________________________
5. ____________________________
6. ____________________________
7. ____________________________

C9. Which bacteria do QC officials focus on?

1. ____________________________
2. ____________________________

3. ______________________________
4. ______________________________
5. ______________________________
6. ______________________________
7. ______________________________

C10. What manual or method is used for detection of *Salmonella*?
____
____

C11. If *Salmonella*/other zoonotic bacterium found to be present on final product, state protocol.
____
____
____

C12. Are labels of final products tagged using an identification system eg. Barcode?
1. Yes 2. ☐ No

C13. If Yes, based on what criteria? E.g. Farm/batch/day processed?
____
____

C14. How often is general decontamination of processing equipment done?
1.☐ Between batches 2.☐ Every other batch
3. ☐ Other(state):____

C15. What equipment are included in general decontamination process? If not all equipment coming into contact with carcasses/effluents of processing, state which are excluded.
1.☐All
2.☐Some: a. ________________________________
b. ________________________________
c. ________________________________
d. ________________________________
e. ________________________________
f. ________________________________

C16. What agents are used for general decontamination? State in order of usage
1. ____________________________________
2. ____________________________________
3. ____________________________________
4. ____________________________________
5. ____________________________________

C17. How often is a thorough decontamination done?
1.☐Overnight 2.☐Every other day 3.☐Once a week 4.☐ Twice a week

C18. What equipment are included in the thorough decontamination process? If not all equipment coming into contact with carcasses/effluents of processing, state which are excluded.
1.☐All
2.☐Some: a. ________________________________
b. ________________________________
c. ________________________________
d. ________________________________
e. ________________________________

f. ___________________________________

C19. What agents are used for the thorough decontamination? State in order of usage

1. ______________________________________
2. ______________________________________
3. ______________________________________
4. ______________________________________
5. ______________________________________

C20. What is the source of water used during processing? ie. Water coming into contact with carcasses e.g. Spraying of carcasses/use in chilled water bath.

____
____
____

C21. Do you treat your water in-house?

1.☐ Yes 2.☐ No

C22. If yes, what chemicals and concentrations are used?

	Agent	Concentration used
1		
2		
3		
4		
5		

SECTION D: Protocols employed

D1. Are workers colour coded (color of coverall) based on the area that they are working? ie Dirty vs clean areas

1.☐ Yes 2.☐ No

D2. If yes, how many areas are selective and which areas?

1	
2	
3	
4	
5	
6	

D3. Attire of workers handling birds/carcasses:

Location	Aprons	Hair net	Gloves			
Pre slaughter						
Initial slaughter/processing						
Final processing						

SECTION E: Products sold to the public

E1. What 'raw' products are made available to consumers? (Whole carcass, parts) Please list.

1	
2	
3	

4	
5	
6	
7	
8	
9	
10	
11	
12	
13	
14	

E2. What further-processing products (cooked) are sold to the public?

1	
2	
3	
4	
5	
6	
7	
8	
9	

E3. Are broilers the only specie processed in this plant? If No, state other species processed
1.☐Yes 2.☐No List: ____

THANK YOU FOR YOUR ASSISTANCE
